# lncRNA DANCR promotes the migration an invasion and of trophoblast cells through microRNA-214-5p in preeclampsia

**DOI:** 10.1080/21655979.2021.1988373

**Published:** 2021-11-29

**Authors:** Qian Zhang, Zhenzhen Wang, Xianghong Cheng, Haiying Wu

**Affiliations:** Department of Obstetrics, Henan Provincial People's Hospital; People's Hospital of Zhengzhou University, Zhengzhou, Henan, 450003, China

**Keywords:** Chorionic trophoblast cells, miR-214-5p, epithelial-mesenchymal transition, lncRNA DANCR, preeclampsia

## Abstract

Studies have shown that lncRNA DANCR is down-regulated in placental tissues of patients with preeclampsia (PE). The aim of this study was to explore the effect of lncRNA DANCR on trophoblast cells as well as its acting mechanism. We disrupted or overexpressed lncRNA DANCR in trophoblast cells HTR-8/SVneo and JEG-3 and detected the associated cellular functional changes by MTT, flow cytometry, Transwell experiment, and scratch experiment. The results showed that overexpression of lncRNA DANCR significantly increased the proliferation, invasion, migration, and EMT process of trophoblast cells. Interfering with lncRNA DANCR showed the opposite result. Further, the targeted interaction between lncRNA DANCR and miR-214-5p was confirmed by the dual-luciferase reporter gene assay. In addition, the expression of PI3K/AKT signaling pathway-related proteins was analyzed by Western blot. Overexpression of lncRNA DANCR can increase the phosphorylation of PI3K/AKT protein and activate this signaling pathway. In conclusion, the enforcing of lncRNA DANCR activates the activation of the PI3K/AKT pathway by down-regulating miR-214-5p, and promotes the migration and invasion of chorionic trophoblast cells. This provides a potential new target for PE therapy.

## Introduction

Preeclampsia (PE), a serious specific complication that occurs during the pregnancy, is an important death cause for pregnant women [1]. The worldwide incidence of PE is 4–5%, while the perinatal incidence of pregnant women is even high to 50% [2]. The main symptoms of PE include high blood pressure and elevated urine protein levels. If not treated timely and effectively, it will lead to central nervous system syndrome, kidney damage, and liver damage [3]. At the same time, PE has a serious adverse effect on the development of the fetus [4]. Studies have shown that it is an indispensable physiological process for the placenta development that the chorionic trophoblast cells have to infiltrate into the muscle layer of the uterine wall, while their migration and invasion ability of PE patients is distinctly decreased, leading to obvious poor growth of the fetus [5]. The combination of labetalol and magnesium sulfate is usually used to treat PE clinically mostly to slow down the progression of the disease, but will prolong the pregnancy period [6]. The pathogenesis of PE involves many aspects, including abnormal oxygen supply, impaired spiral arterial remodeling, and maternal blood vessel destruction [7]. At the same time, many studies have pointed out that PE is caused by the abnormal expression of many genes related to PE [8]. Therefore, we can learn more about the biological mechanism of PE by investigating the biomarkers related to the onset of PE, thus providing more options for diagnosing and treating the disease.

Long non-coding RNA (lncRNA) is an RNA with no protein-coding ability and longer than 200 nt [9]. There are various approaches for LncRNAs to regulate gene expression, for instance, chromatin modification, post-transcriptional modification, and transcription [10]. In addition, lncRNA can also affect the expression of related genes by competing with microRNAs (miRNAs) at the horizontal level after transcription, thereby regulating the occurrence and development of related diseases [11]. MiRNA belongs to the non-coding RNA whose length was about 20 nt, which can specifically target and combine with the 3ʹ-UTR region of the mRNA, thereby inhibiting mRNA translation and its expression [12]. It has been testified that lncRNA has participated in various pathological and biological processes, such as cell metabolism, immune response, cell differentiation, and disease development [13,14], and abnormal lncRNAs levels can promote or inhibit the development of diseases [15]. With the development of high-throughput sequencing, it has been found that lncRNAs can be used as biomarkers in different human diseases [16], and many scholars have found that they are the key to PE development. Cao et al. has confirmed that lncRNA Uc.187 expresses evidently higher in PE patients, which led to the abnormal biological behavior of HTR-8/SVneo cells, thereby promoting the development of PE [17]. Zhang et al. [18] found that lncRNA FOXD2-AS1 expresses low in the serum of PE patients, and through down-regulating the miR-3127 expression, it can suppress the migration, invasion, and proliferation of trophoblast cells. Gong et al. [19] reported that lncRNA TDRG1 can negatively regulate miR-214-5p, thereby promoting cell invasion and proliferation. All the above suggests that lncRNAs may exert a crucial function in PE occurrence and development. Some articles revealed that up-regulated lncRNA DANCR expression is related to enhancing the cell invasion and migration ability [20]. Also, in severe PE, the lncRNA DANCR expression was significantly reduced and could be involved in PE pathogenesis by regulating the proliferation and infiltration of trophoblasts [21]. However, the specific mechanism of lncRNA DANCR affecting PE pathogenesis has not been studied in depth. Bioinformatics predicts that lncRNA DANCR binds to miR-214-5p. Moreover, miR-214-5p was testified to be highly expressed in PE placental tissues [22]. However, the role of miR-214-5p and lncRNA DANCR in PE remains unclear. Therefore, the purpose of this experiment was to study the connection between miR-214-5p and lncRNA DANCR, and to explore the influence of lncRNA DANCR/miR-214-5p axis upon chorionic trophoblast cell function and its mechanism, in order to provide an effective theoretical basis for studying the pathogenesis of PE.

## Materials and methods

### Cell culture

We purchased the human chorionic trophoblast cells HTR-8/SVneo and human chorionic carcinoma cells JEG-3, all cultured in a complete RPMI-1640 medium, from the American Type Culture Collection (ATCC), and the medium includes 10 mg/mL streptomycin (Gibco, USA), 10% fetal bovine serum (FBS, Gibco, USA), and 100 mg/mL penicillin, which was then settled in an incubator (Thermo, USA) at 37°C and 5% CO_2_.

### Cell transfection

During the logarithmic growth phase, the JEG-3 and HTR-8/SVneo cells were planted on a plate with 6 wells at 1 × 10^5^ cells/well till they were grown and confluent to 60–70%. Following the lipo 3000 transfection kit (Thermo, USA) instructions, the miR-214-5p mimics, DANCR-shRNA interference plasmid (sh-DANCR), and negative control shRNA plasmid (sh-NC), mimics NC, and pc-DNA3.1-DANCR overexpression plasmid (DANCR) and control plasmid pc-DNA3.1 (pc-NC) were transfected into the cells, respectively, and then placed in the incubator for 48 h at 37°C and 5% CO_2_ before proceeding to follow-up experiments.

### Quantitative real-time polymerase chain reaction (qRT-PCR)

The total RNA from cells was extracted with 1 mL TRizol reagent (Invitrogen, USA). Subsequently, NanoDrop was used for determining the concentration and purity of RNA, and cDNA was prepared by the random primer reverse transcription kit (Thermo, USA). 2 µl cDNA was used in a 20 µl qPCR reaction. The miR-214-5p and lncRNA DANCR expression levels were evaluated by the SYBR GREEN kit (TaKaRa, Japan). The cycling condition was: 95°C for 10 min and 40 cycles of 95°C for 10 s, and 58°C for 30 s. GAPDH and U6 were selected as internal reference for lncRNA and miRNA. The qRT-PCR experimental data were counted following the 2^−ΔΔCt^ method to determine the target gene expression. The following Table 1 include the primer sequences.

**Table 1.** Primer sequences

RNA	Sequences(5ʹ to 3ʹ)
miR-214-5p	F: ACACTCCAGCTGGGCGTGTCGTTCACATCT
	R: CUACAAAGGGAAGCGACAGGCA
lncRNA DANCR	F: GCCACAGGAGCTAGAGCAGT
	R: GCAGAGTATTCAGGGTAAGGGT
GAPDH	F: CATCACTGCCACCCAGAAGACTG
	R: ATGCCAGTGAGCTTCCCGTTCAG
U6	F: CTCGCTTCGGCAGCACAT
	R: TTTGCGTGTCATCCTTGCG

### MTT detection

After transfection, during the logarithmic growth phase, JEG-3 or HTR-8/Svneo cells were planted to a plate with 96 wells at 5 × 10^3^ cells/well, and then they were, respectively, cultured for 24, 48, and 72 h. We transferred 20 μL MTT solution of 5 mg/mL to each group of cells, and cultured them for 4 h in an incubator. Later, the supernatant was removed, DMSO of 150 μL was transferred inside, and then was shaken 15 min. In final, by using a microplate reader, the absorbance value at 570 nm was counted.

### Cell cycle

JEG-3 or HTR-8/Svneo cells in logarithmic growth phase were planted in a 6-well plate at 1 × 10^6^ cells/well. After transfection for 48 h, the cells were collected and fixed overnight at 4°C with pre-cooled 70% ethanol. On the next day, the fixed cells were collected and resuspended by adding 400 μL PBS including 100 μg/ml RNAase and 100 μg/ml PI staining solution (Thermo, USA). After staining for 30 min at the room temperature in the dark, the cells were assayed and analyzed using a BD FACSCalibur flow cytometer (BD Biosciences, USA).

### Transwell detection

The transwell chamber (Corming, USA) was coated with matrix gel at 37°C for 30 min, then the cell suspension of 100 μL to be tested was put into the upper chamber, with 700 μL medium which contains 20% FBS into the lower chamber. After incubating at 5% CO_2_ and 37°C for 12–24 h, we took out the Transwell chamber, rinsed it with PBS 3 times, and fixed it by 1% glutaraldehyde for 30 min. Later, it was washed by PBS, dried, and soaked by 0.1% crystal violet for 12 h. Then, it was washed again with PBS, and we observed it with an upright microscope after its drying. We chose 6–10 fields randomly, all of whose positive cell numbers were recorded and 3 of which were taken pictures and analyzed.

### Scratch test

First, on the back of the 6-well plate, a horizontal line was painted by a marker pan along with a ruler, and then transfected cells were planted to the plate and cultivated until the confluence to 60–70%. A sterilized pipette tip 10 μL was put perpendicular to the horizontal line to draw line scratches. PBS was used for discarding cell debris and suspended cells, and later the rest were put in a fresh serum-free medium and cultured for 24 h. An inverted microscope was employed to take pictures, with the recorded results, and the scratch area was evaluated by ImageJ software.

### Dual-luciferase reporter gene experiment

The potential-binding sequence of miR-214-5p and lncRNA DANCR was predicted by ENCORI. The binding sequence as well as its mutant sequence were inserted into the pGL3 vector (Promega, USA) to create the lncRNA DANC-WT and lncRNA DANCR-MUT reporter vectors. Then mimics NC and miR-214-5p mimics were, respectively, mixed with lncRNA DANCR-MUT and lncRNA DANCR-WT recombinant plasmids and were transfected into 293 T cells through Lipo2000 liposomes. After 48 hours of transfection, by using the reporter gene detection kit, the luciferase activity was determined (Promega, USA).

### Western blot

Cells were collected and total protein were extracted using cell lysate (Thermo, USA), and the concentration of proteins was evaluated by a BCA kit (Beyotime, China). The proteins (20 μg) were separated by 10% SDS-PAGE and then transferred to the polyvinylidene difluoride (PVDF) membranes (Millipore, MA). After blocking with 5% skimmed milk for 1 h at room temperature, the membranes were incubated with the primary antibodies overnight at 4°C. Then it was rinsed three times and incubated with an HRP-conjugated secondary antibody at room temperature for 1 h. After washing the membrane three more times, we applied a chemiluminescence reagent for developing the proteins, which were placed under the gel imaging system for the image collection, and the gray value of the protein band was analyzed by ImageJ software for the calculation of the protein expression. The primary antibodies used in the study were as follows: E-cadherin (#14472, CST, USA), Vimentin (#5741, CST), N-cadherin (#13116, CST), Twist (ab50887, Abcam, UK), p-PI3K (ab278545, Abcam), PI3K (ab191606, Abcam), p-AKT (ab38449, Abcam), AKT (ab38449, Abcam), and GAPDH (ab8245, Abcam). The internal control was GAPDH.

### Statistical analysis

All statistical analyses were performed using the SPSS 26.0 software. All data were presented as mean ± standard deviation (SD). Student’s t-tests or one-way analysis of variance (ANOVA) for normally distributed data were applied to evaluate the difference between two groups or multiple groups. P < 0.05 was used as the criterion for judging the significance of the difference.

## Results

### Promotion of the proliferation and cell cycle of trophoblast cells by up-regulated lncRNA DANCR

**Figure 1.**
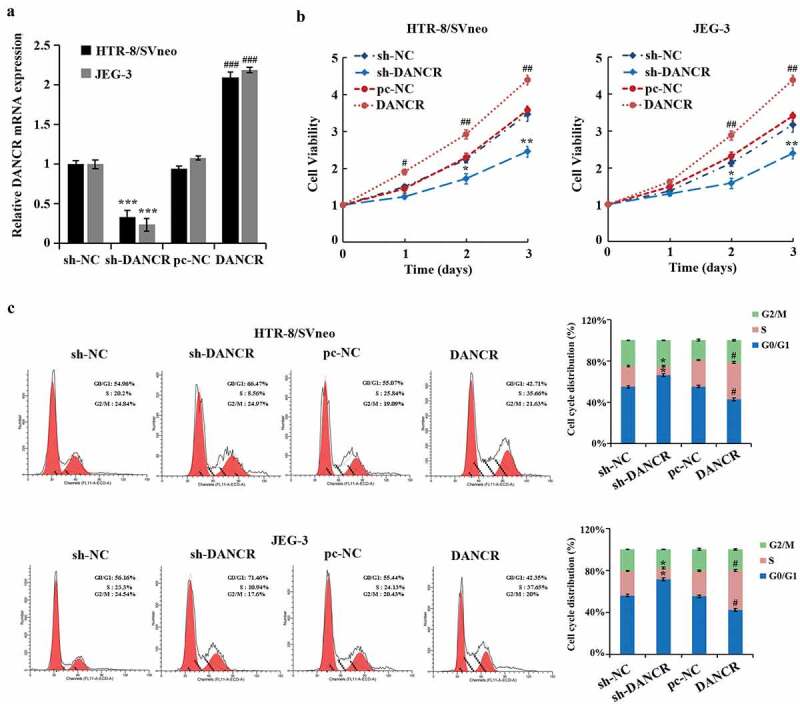
The effect of up-regulating the expression of lncRNA DANCR on the trophoblast cell cycle and cell proliferation

To further study the effect of lncRNA DANCR on the function of trophoblast cells, we evaluated the lncRNA DANCR expression after interference with or overexpression in trophoblast cells JEG-3 and HTR-8/SVneo cells, suggesting that lncRNA DANCR expression in the sh-DANCR group was distinctly lower than the sh-NC group. However, it was markedly higher than the pc-NC group (P < 0.001), exhibiting a successful transfection (Figure 1a). After silencing the lncRNA DANCR, the proliferation rates of JEG-3 and HTR-8/SVneo cells were evidently decreased (P < 0.01, Figure 1b), and the proportion of cells during the G0/G1 phase was notably higher, while during the S phase was markedly lower (P < 0.05, Figure 1c), indicating that the cell cycle was contained to G1 phase. When overexpressing the lncRNA DANCR, the results were totally opposite. These results exhibited that up-regulated lncRNA DANCR promoted the proliferation and cell cycle of trophoblast cells.

(a) qRT-PCR for evaluating the efficiency of overexpressing (pc-NC, DANCR) and interfering with the lncRNA DANCR (sh-NC, sh-DANCR); (b) determination of the cell proliferation of each group of cells using MTT; (c) measurement of cell cycles in each group using flow cytometry; ^#^*p* < 0.05, ^##^*p* < 0.01 and ^###^*p* < 0.001 vs ., pc-NC group. *p < 0.05, **p < 0.01 and ***p < 0.001 vs., sh-NC group.

### Promotion of the trophoblast cells transferring by Overexpressing lncRNA DANCR

**Figure 2.**
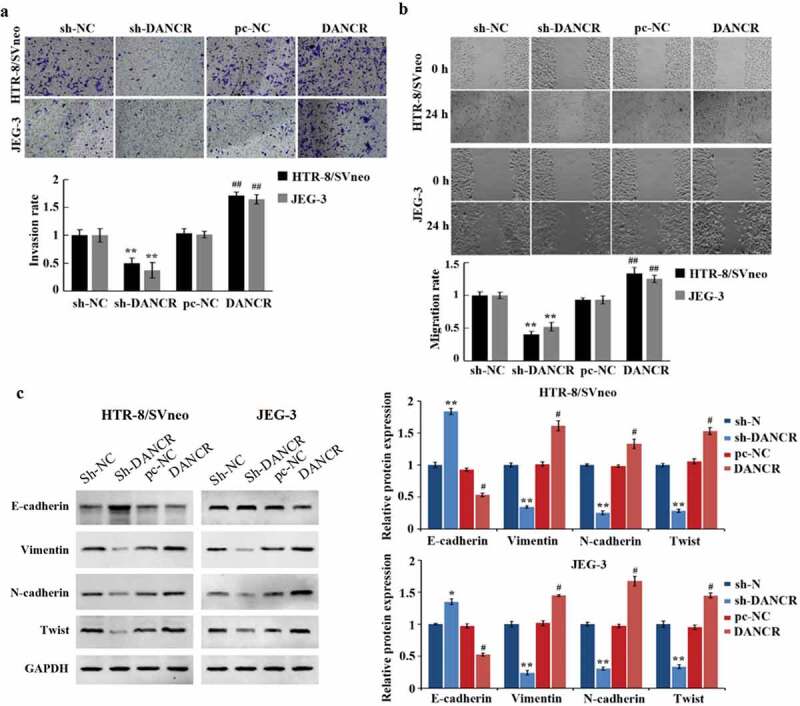
Up-regulation of lncRNA DANCR can promote the migration, invasion, and epithelial-mesenchymal transition of trophoblast cells

Cell migration and invasion are important characteristics of PE development. Transwell experiment, scratch experiment, and Western blot experiment were used to measure the influence of lncRNA DANCR on the migration, invasion, and EMT of trophoblast cells. And it has been proved that overexpressing lncRNA DANCR can promote the migration and invasion of JEG-3 and HTR-8/SVneo cells (P < 0.01), while the E-cadherin expression was markedly reduced (P < 0.05), and the N-cadherin, Twist, and Vimentin were largely increased (P < 0.05), which promoted the EMT process (Figure 2a–c). While we silenced lncRNA DANCR, we obtained the opposite results. These results indicated that the up-regulation of lncRNA DANCR can accelerate the process of PE.

(a) Assessment of the invasion ability using Transwell; (b) scratch test for observing the migration ability; (c) Western blot for determining the E-cadherin, vimentin, N-cadherin, and Twist expression; ^#^*p* < 0.05 and ^##^*p* < 0.01 vs., pc-NC group. *p < 0.05 and **p < 0.01 vs., sh-NC group.

### MiR-214-5p is directly targeted by LncRNA DANCR to regulate its expression

**Figure 3.**
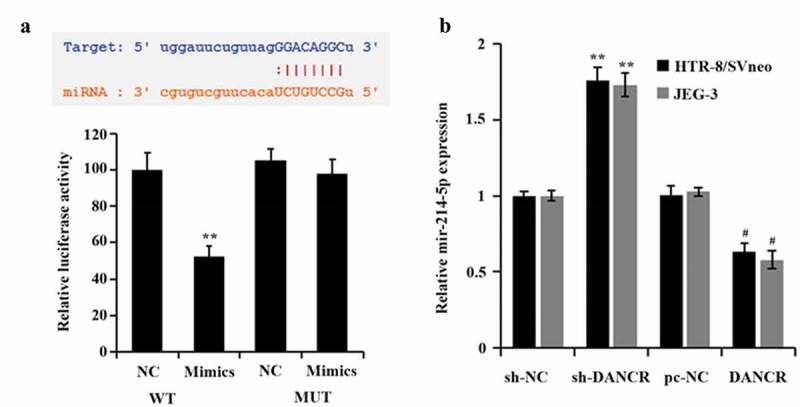
Connection of miR-214-5p and lncRNA DANCR

In order to explore how lncRNA DANCR regulates the PE process, we predicted the potential targets of lncRNA DANCR through the bioinformatics website ENCORI (http://starbase.sysu.edu.cn/). Finally, it was found that lncRNA DANCR and miR-214-5p had an interaction sequence (Figure 3a). Then, the dual-luciferase reporter gene experiment also proved that the luciferase activity of the lncRNA DANCR-WT vector was evidently suppressed by miR-214-5p (P < 0.01), which had no influence on the lncRNA DANCR-MUT vector (Figure 3a). In addition, when lncRNA DANCR was overexpressed in trophoblast cells, the miR-214-5p expression decreased distinctly (P < 0.05), while its expression increased when lncRNA DANCR was suppressed (P < 0.01, Figure 3b), indicating that miR-214-5p was negatively regulated by lncRNA DANCR through straightly targeting it.

(a) Verification of the targeted interaction of miR-214-5p and lncRNA DANCR by dual-luciferase assay, **p < 0.01 vs., NC group; (b) qRT-PCR for evaluating the miR-214-5p expression in trophoblast cells, ^#^*p* < 0.05 vs., pc-NC group, **p < 0.01 vs., sh-NC group.

### LncRNA DANCR regulates the cell cycle, migration, invasion, proliferation, and EMT of trophoblast cells by miR-214-5p

**Figure 4.**
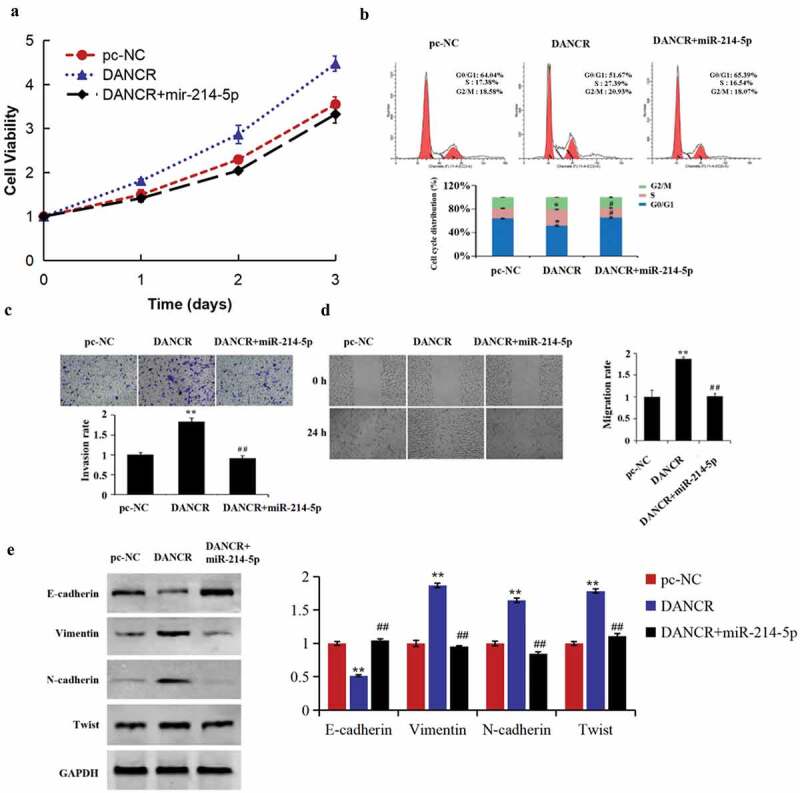
lncRNA DANCR regulates the preeclampsia progress by binding to miR-214-5p

For studying how miR-214-5p and lncRNA DANCR function during the PE progress, we conducted a cell recovery experiment. The results demonstrated that up-regulating miR-214-5p reversed the promoted function of lncRNA DANCR overexpression on the cell cycle, migration, invasion, and proliferation of HTR-8/SVneo cells (Figure 4a–d). Meanwhile, EMT-related protein expressions in the DANCR group were reversed by miR-214-5p mimics (Figure 4e), revealing that lncRNA DANCR regulated the PE progress by competitively binding to miR-214-5p.

(a) MTT for determining the proliferation of HTR-8/SVneo cells; (b) flow cytometry for assessing the cell cycle of HTR-8/SVneo cells; (c) measurement of the invasion ability of HTR-8/SVneo cells by Transwell experiment; (d) scratch test for evaluating the migration ability of HTR-8/SVneo cells; (e) Western blot for assessing the EMT-related protein expression; *p < 0.05 and **p < 0.01 vs., pc-NC group, ^#^*p* < 0.05 and ^##^ *p* < 0.01 vs., DANCR group.

### Activation of the PI3K/AKT pathway by lncRNA DANCR

**Figure 5.**
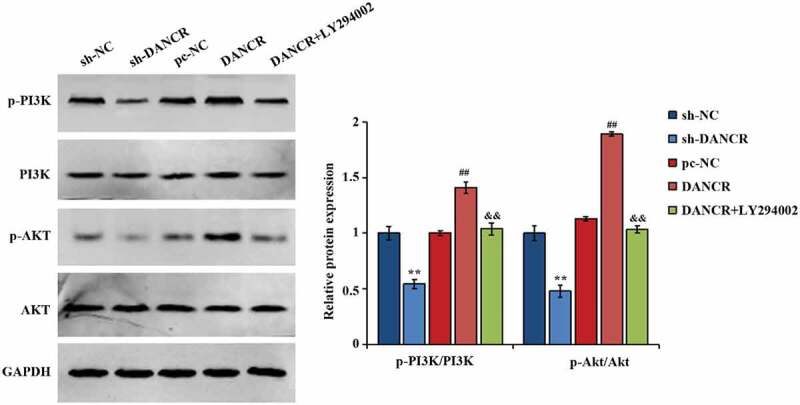
The function of lncRNA DANCR on proteins related to PI3K/AKT pathway

Studies have shown that the AKT pathway exerts some function in the pathogenesis of PE, and downregulating p-AKT in trophoblast cells can affect cell invasion and proliferation [23,24]. Thus, the interaction of lncRNA DANCR and PI3K/AKT pathways was further studied, and found that when lncRNA DANCR was overexpressed, the p-PI3K and p-AKT expression in cells raised largely, and ratios of p-PI3K/PI3K and p-AKT/AKT also raised greatly. After adding the PI3K inhibitor LY294002, the p-PI3K, and p-AKT expression in the cells were evidently lower, and the ratios of p-AKT/AKT and p-PI3K/PI3K were obviously suppressed (P < 0.01, Figure 5), exhibiting that lncRNA DANCR can significantly affect the function of PI3K/AKT pathway.

&&*p* < 0.01 vs., DANCR+LY294002 group, ^##^*p* < 0.01 vs DANCR group, **p < 0.01 vs., pc-NC group.

## Discussion

PE can cause fetal death, fetal dysplasia, and damage to different organs of the mother [25]. In recent years, a large number of studies have continued to study PE, but the exact reason has not been elucidated [26]. Therefore, it is necessary to explore its internal mechanism and formulate new treatment strategies. It has been revealed that in the PE process, many lncRNAs exert an indispensable function. For instance, in the PE process, MEG3 and lncRNA SPRY4-IT1 regulate the apoptosis and proliferation of trophoblast cells, as well as the migration, invasion, and formation of blood vessels [27,28]; meanwhile, lncRNA RPAIN can regulate the trophoblast cell apoptosis in PE [29].

Dysfunction of helical artery remodeling on the surface of maternal placenta and insufficient invasion of extravasation trophoblastic layer are the key factors causing PE [30,31]. The abnormal cell migration and invasion ability of extravillous trophoblast cells (EVTs) is considered to be an important factor in PE development [24]. EMT is necessary for cell migration and invasion, and it is characterized by the low-expressed key epithelial markers (E-cadherin) and high-expressed mesenchymal markers (β-catenin and vimentin) [32,33], among which the E-cadherin expression is negatively connected with the cell EMT, while both Vimentin and N-cadherin expressions are positively interacted with the cell EMT level [34]. In this study, we found that up-regulating the expression of lncRNA DANCR can promote the migration, proliferation, invasion, and EMT process of trophoblast cells. According to all the results above, it can be acknowledged that by inducing EMT process, up-regulated lncRNA DANCR can promote the migration, invasion, and proliferation of trophoblast cells. This is similar to the findings of Tang et al. who found that lncRNA PROX1-AS1 could mediate placental trophoblast migration and invasion through the miR-211-5p/caspase-9 axis, thereby slowing the progression of PE [35].

Studies have shown that the approach for lncRNA to exert its biological functions is mainly through binding microRNAs to regulate downstream pathways. Among them, miR-214-5p has been found to have the biological effects of inhibiting cell proliferation, invasion, and migration. Pang et al. found that miR-214-5p can target the KLF5 gene, inhibit the liver cancer cell’s migration and proliferation, and contain its cell cycle to the G0/G1 phase [36]. Zheng et al. [37] discovered that miR-214-5p can regulate CRMP5 and inhibit the growth, migration, as well as colony formation of prostate cancer cells, additionally, it also contains cells to G0/G1 phase and promotes cell apoptosis. In this study, miR-214-5p was confirmed to bind to lncRNA DANCR. It was further discovered that over-expressed miR-214-5p reversed the influence of lncRNA DANCR up-regulation on the cell cycle, migration, invasion, and proliferation of trophoblast cells, this is consistent with the results of previous studies [19]. Meanwhile, researches have exhibited that miR-214-5p can target the Jagged1 to inhibit the migration, invasion, and proliferation of trophoblast cells in PE [22]. However, the role of miR-214-5p in lncRNA DANCR affecting PE development was not investigated in this study.

Phosphatidylinositol-3-kinase (PI3K)/AKT pathway exerts a critical function in keeping the biological characteristics of malignant cells and regulating cell proliferation [38]. Activating PI3K can create PIP3 in the plasma membrane by tyrosine kinases. What causes the AKT accumulation on the membrane is the interaction of PIP3 and the PH domain of AKT. Meanwhile, 3-phosphoinositide-dependent protein kinase 1 (PDK1) is able to phosphorylate Thr308 of AKT, thereby activating the AKT, and all these changes may trigger EMT [39]. The process of PI3K/AKT pathway mediating EMT has attracted extensive notice on potential targets for preventing and treating metastatic tumors [40]. At the same time, a study has shown that the there is an interaction of PI3K/Akt/mTOR pathway and trophoblasts cell behavior [23], and down-regulating DDX46 can suppress the migration and proliferation of PE trophoblasts through the PI3K/Akt/mTOR pathway [41]. In this study, it was also found that lncRNA DANCR can significantly raise the phosphorylation level of PI3K and AKT in cells, discovering that through activating the PI3K/AKT pathway, lncRNA DANCR may promote the EMT process of trophoblast cells.

## Conclusion

In summary, lncRNA DANCR mediates trophoblast proliferation, migration, invasion, and EMT by activating the PI3K/AKT pathway and up-regulating the miR-214-5p expression. It is suggested that lncRNA DANCR can be chosen as a new treating target and is a potential biomarker for PE. However, the function of lncRNAs is very complicated, and this study failed to fully clarify the function of lncRNA DANCR. Therefore, further in vivo experiments and mechanism exploration are needed to uncover the PE pathogenesis in depth and give greater contributions to curing the disease.

## Highlights

1. lncRNA DANCR increases the proliferation, invasion, migration, and EMT process of trophoblast cells.
2. lncRNA DANCR binds to miR-214-5p.

lncRNA DANCR increases the phosphorylation of PI3K/AKT protein.
